# Analysis of common genetic variation and rare CNVs in the Australian Autism Biobank

**DOI:** 10.1186/s13229-020-00407-5

**Published:** 2021-02-10

**Authors:** Chloe X. Yap, Gail A. Alvares, Anjali K. Henders, Tian Lin, Leanne Wallace, Alaina Farrelly, Tiana McLaren, Jolene Berry, Anna A. E. Vinkhuyzen, Maciej Trzaskowski, Jian Zeng, Yuanhao Yang, Dominique Cleary, Rachel Grove, Claire Hafekost, Alexis Harun, Helen Holdsworth, Rachel Jellett, Feroza Khan, Lauren Lawson, Jodie Leslie, Mira Levis Frenk, Anne Masi, Nisha E. Mathew, Melanie Muniandy, Michaela Nothard, Peter M. Visscher, Paul A. Dawson, Cheryl Dissanayake, Valsamma Eapen, Helen S. Heussler, Andrew J. O. Whitehouse, Naomi R. Wray, Jacob Gratten

**Affiliations:** 1 Mater Research Institute, The University of Queensland, Brisbane, QLD Australia; 2 Institute for Molecular Bioscience, The University of Queensland, Brisbane, QLD Australia; 3 Cooperative Research Centre for Living With Autism (Autism CRC), Long Pocket, Brisbane, QLD Australia; 4 Telethon Kids Institute, The University of Western Australia, Perth, WA Australia; 5 Max Kelsen, Fortitude Valley, QLD Australia; 6 School of Psychiatry, University of New South Wales, Sydney, NSW Australia; 7 Child Health Research Centre, The University of Queensland, Brisbane, QLD Australia; 8 Olga Tennison Autism Research Centre, La Trobe University, Melbourne, VIC Australia; 9 Queensland Brain Institute, The University of Queensland, Brisbane, QLD Australia; 10 Academic Unit of Child Psychiatry South West Sydney, Ingham Institute, Liverpool Hospital, Sydney, NSW Australia; 11 Child Development Program, Children’s Health Queensland, Brisbane, QLD Australia

**Keywords:** Autism spectrum disorder, Genetics, Polygenic score, Copy number variation, Australian autism biobank

## Abstract

**Background:**

Autism spectrum disorder (ASD) is a complex neurodevelopmental condition whose biological basis is yet to be elucidated. The Australian Autism Biobank (AAB) is an initiative of the Cooperative Research Centre for Living with Autism (Autism CRC) to establish an Australian resource of biospecimens, phenotypes and genomic data for research on autism.

**Methods:**

Genome-wide single-nucleotide polymorphism genotypes were available for 2,477 individuals (after quality control) from 546 families (436 complete), including 886 participants aged 2 to 17 years with diagnosed (*n* = 871) or suspected (*n* = 15) ASD, 218 siblings without ASD, 1,256 parents, and 117 unrelated children without an ASD diagnosis. The genetic data were used to confirm familial relationships and assign ancestry, which was majority European (*n* = 1,964 European individuals). We generated polygenic scores (PGS) for ASD, IQ, chronotype and height in the subset of Europeans, and in 3,490 unrelated ancestry-matched participants from the UK Biobank. We tested for group differences for each PGS, and performed prediction analyses for related phenotypes in the AAB. We called copy-number variants (CNVs) in all participants, and intersected these with high-confidence ASD- and intellectual disability (ID)-associated CNVs and genes from the public domain.

**Results:**

The ASD (*p* = 6.1e−13), sibling (*p* = 4.9e−3) and unrelated (*p* = 3.0e−3) groups had significantly higher ASD PGS than UK Biobank controls, whereas this was not the case for height—a control trait. The IQ PGS was a significant predictor of measured IQ in undiagnosed children (*r* = 0.24, *p* = 2.1e−3) and parents (*r* = 0.17, *p* = 8.0e−7; 4.0% of variance), but not the ASD group. Chronotype PGS predicted sleep disturbances within the ASD group (*r* = 0.13, *p* = 1.9e−3; 1.3% of variance). In the CNV analysis, we identified 13 individuals with CNVs overlapping ASD/ID-associated CNVs, and 12 with CNVs overlapping ASD/ID/developmental delay-associated genes identified on the basis of de novo variants.

**Limitations:**

This dataset is modest in size, and the publicly-available genome-wide-association-study (GWAS) summary statistics used to calculate PGS for ASD and other traits are relatively underpowered.

**Conclusions:**

We report on common genetic variation and rare CNVs within the AAB. Prediction analyses using currently available GWAS summary statistics are largely consistent with expected relationships based on published studies. As the size of publicly-available GWAS summary statistics grows, the phenotypic depth of the AAB dataset will provide many opportunities for analyses of autism profiles and co-occurring conditions, including when integrated with other omics datasets generated from AAB biospecimens (blood, urine, stool, hair).

## Background

Autism spectrum disorder (ASD) is a neurodevelopmental condition with significant clinical heterogeneity. Heritability estimates are high (~ 80%), and the genetic architecture is complex, involving de novo, rare and common genetic variants [1]. One approach to disentangle this heterogeneity and understand how genetics contribute to autism-associated clinical features is through cohort datasets that combine deep phenotypic information with biological datasets.

In autism, the majority of the genetic variation tracked by heritability is accounted for by common genetic variation [2, 3], as quantified by the single nucleotide polymorphism (SNP)-based heritability which is estimated to be ~ 40–50% [2]. As with other common neuropsychiatric conditions, common variants contributing to autism have small effect sizes, requiring large sample sizes for identification. The largest published ASD genome-wide association study (GWAS) [4] identified five genome-wide significant loci in an analysis of 18,381 cases and 27,969 controls, and an additional seven loci were identified using multi-trait analysis of GWAS [5] methodology leveraging power from correlated traits. Larger sample sizes will be necessary to identify further loci, which are expected to be found given the substantial estimate of SNP-based heritability [2].

De novo and rare inherited variants contribute to ~ 10% of diagnoses of autism [1]. They include karyotype abnormalities [6], copy number variants [7] (CNVs) including deletions and duplications, and deleterious point mutations such as loss-of-function and missense variants [8, 9]. De novo genetic variants predominantly do not contribute to heritability estimates, which only track genetic features shared by relatives. CNVs are of particular interest in ASD with rare, large CNVs being associated with ASD, as well as commonly co-occurring conditions such as developmental delay (DD) and intellectual disability (ID). De novo CNVs occur at three to five times the rate of controls or unaffected siblings [9, 10], tend to have modest to large effect sizes [11, 12], and the number of affected genes is associated with autism propensity [9]. Although many forms of rare variation require sequencing approaches to detect, larger CNVs may be detected from genome-wide SNP arrays using software such as PennCNV [13] and iPattern [10]. This is useful as it enables analysis of CNVs in ASD studies which include genotyping data.

The Australian Autism Biobank (AAB) [14] is an initiative of the Australian Cooperative Research Centre for Living with Autism (Autism CRC, website at [15]) that recruited individuals aged 2 to 17 years diagnosed with ASD, their parents and undiagnosed siblings, together with unrelated undiagnosed children from Australia’s four most populous states. The value of the AAB, although relatively modest in size, lies in the depth of the dataset, which includes detailed phenotypic data (psychological and behavioural testing, medical history, parental medical history, and lifestyle data including diet (children only), parental occupation and educational attainment, and parental exposures to psychological and chemical hazards), biospecimens (including blood, urine, stool, hair and saliva) and derived genomic data (SNP genotyping, DNA methylation, stool metagenomics, and metabolomics). This combination of biological data and deep phenotyping offers an opportunity to dissect the clinical and genetic heterogeneity inherent in autism.

In particular, the depth of data in the AAB lends itself to prediction analyses. Polygenic scoring methodology enables prediction of phenotypes based on common genetic markers, based on the assumption that complex traits are underpinned by many additive variants of small effect (which are on the liability scale for disease traits). Polygenic scores (PGS) leverage summary statistics from independent large-scale GWAS to obtain effect-sizes of trait-associated alleles. Applied to an individual, the effect-sizes for a given allele are multiplied by that individual’s allele dosage, and summed over all alleles to calculate their PGS. Thus, for a given complex trait, an individual’s PGS estimates propensity for that trait, or at least that which is captured by common genetic variation. For disease traits, the PGS are usually called polygenic risk scores, with higher values implying higher risk for the disease.

Here, we present analyses using genome-wide SNP data from a total of 2,477 individuals in the AAB. First, we use these genetic data to confirm relationships and ancestry. Second, we leverage publicly-available summary statistics for ASD, IQ and other traits to explore the common genetic basis of autism in the AAB, together with a variety of phenotypes including psychometric testing results and diagnostic features. Third, we call CNVs and identify overlap with publicly-available, curated lists of CNVs and genes that have been associated with ASD, ID and DD.

## Methods

### Overview of dataset

A full description of the AAB biobank is provided by Alvares et al. [14]. Participants were recruited between 2013 and 2018 from four major autism research centres or developmental clinics across Australia—in Perth, Brisbane, Melbourne and Sydney. AAB participants fell into four groups: 1) child participants (aged 2–17 years) diagnosed with ASD or queried for the condition (hereafter referred to as “ASD”), with some multiply-affected families; 2) parents of children with ASD; 3) siblings (“SIB”) of diagnosed children who did not themselves meet ASD criteria; and 4) unrelated children (“UNR”) without an ASD diagnosis and with no known first-degree relatives with an ASD diagnosis. The UNR group was recruited from the community: predominantly from children (Generation 3) of the Generation 2 participants from the Raine Study in Perth [16], and through community advertising and in association with health service providers in the other centres. This group did not have exclusion criteria other than ASD diagnosis (i.e., there were no exclusions for other psychiatric, medical or genetic conditions, cognitive function, or medication use). Detailed phenotypic data (see below) and multiple biological samples (blood, stool, urine, saliva, hair) were collected from most (but not all) participants.

### Phenotype data

The AAB includes detailed phenotypic data [14]. For the ASD group, clinical assessments were administered and questionnaires were completed by parents or caregivers, including the Autism Diagnostic Observation Schedule-2 (ADOS-2) [17] or Autism Diagnostic Observation Schedule-G (ADOS-G) [17], Developmental, Dimensional and Diagnostic Interview [18]), Vineland Adaptive Behavior Scale-II [19], and the Short Sensory Profile-2 (SSP-2) [20]. Parents and the SIB and UNR groups completed age-appropriate questionnaires for broader autism spectrum symptoms (Parents: Communication Checklist-Adult [21]; SIB/UNR: Social Responsiveness Scale [22]). Cognitive functioning and IQ in the ASD, SIB and UNR groups was assessed using the Mullen Scales of Early Learning (MSEL [23]) or Wechsler Intelligence Scale for Children 4th edition (WISC-IV) [24], and in parents using the matrix reasoning subtest of the Wechsler Abbreviated Scale of Intelligence 2nd edition (WASI) [25]. Sleep problems in the ASD, SIB and UNR groups were assessed with the Children’s Sleep Habits Questionnaire (CSHQ) [26]. Morphometric measures (height, weight, head circumference) were collected on all participants, and the dataset also includes medical history of parents and the child (including gestational history, immune disorders and gastrointestinal conditions), developmental milestones and early-life exposures.

We used the following summary scores from the psychometric questionnaires: the ADOS-2 Calibrated Severity Score (scored from 1 to 10 indicating the level of ASD-related symptoms compared to children with ASD of the same age and language ability, from ADOS-2 Modules 1–4), the Social Responsiveness Scale total t-score, the CSHQ raw score (sum of items, after reversing relevant questionnaire items), and a CSHQ composite score based on variables that were relevant to chronotype (items: the Sleep Onset Delay, Sleep Duration, Night Waking and Daytime Sleepiness subscales, in addition to the “goes to bed at same time” item of the Bedtime Resistance subscale), the Communication Checklist-Adult z-score, the WISC-IV composite score, the WASI matrix reasoning t-score, and the MSEL non-verbal developmental quotient.

### SNP genotyping quality control (QC) and imputation

SNP genotyping was initially performed on a total 2,491 AAB participants (with 2,477 remaining after QC steps described below) who provided a blood sample, using the Illumina Global Screening Array v1 and v2. The GenomeStudio v2.0.4 software was used to call genotypes and filter low quality samples (call rate ≥ 95%) and SNPs (cluster separation ≥ 0.4, AB R mean ≥ 0.2, AB T mean 0.2 ≤ *x* ≤ 0.8, and GenTrain score ≥ 0.68), prior to strand alignment and standard quality control procedures performed in PLINK1.9 [27, 28]. We excluded *n* = 1 sample with missingness > 0.1 and SNPs with genotyping rate < 0.02, Hardy Weinberg equilibrium test < 1e−6 and/or minor allele frequency < 0.01. We removed duplicated samples, retaining the sample with the lower missingness rate. We also cross-referenced allele frequencies to the Haplotype Reference Consortium reference, excluding SNPs where the difference in minor allele frequency between the AAB dataset and Haplotype Reference Consortium was greater than 0.2.

To help identify sample mix-ups, we checked for discrepancies between reported and genetic sex. In the process of performing these checks, we identified two individuals with sex chromosome aneuploidies (one with diagnosed Turner’s syndrome (XO), and one with Klinefelter’s syndrome (XXY) that had not been recorded in the AAB medical history), and *n* = 4 pairs (total *n* = 8 individuals) of putative sample mix-ups, which were subsequently checked and corrected based on genetic relationships, and manifest sample proximity. We identified a further 12 individuals whose genetic sex based on X chromosome F coefficients of inbreeding (F coefficient) was inconclusive. For these individuals, we calculated genome-wide inbreeding coefficients, and rescued from exclusion female samples with X chromosome *F* < 0.25 and genome-wide inbreeding coefficient ≥ 0.05, as this suggests that the higher X chromosome F coefficient may reflect some degree of consanguineous ancestry. Overall, *n* = 2 samples were excluded on the basis of sex checks.

Imputation to the Haplotype Reference Consortium [29] was performed using the Sanger Imputation Service, with pre-phasing performed using EAGLE2 software [30] which best accommodates the presence of offspring-mother-father trios in the data. The imputed SNPs underwent further QC (excluding SNPs with Hardy–Weinberg equilibrium *p* < 1e-6, minor allele frequency < 0.01, info score < 0.8), leaving a total of 2,478 participants and 7,068,672 SNPs (6,991,521 autosomal markers and 77,151 on chromosome X) for association and prediction analyses.

From called CNVs (below) we identified one child in the UNR group with a CNV diagnosis of Smith-Magenis syndrome, which is associated with DD and can present with autistic features. For this reason, this participant was excluded from subsequent analyses, after which data from 2,477 participants remained in the QC-ed dataset.

### Ancestry assignment and genetic relationships

We inferred genetic ancestry for each individual by projecting the AAB genotyping data onto the first two principal components (PCs) of the 1000 Genomes reference dataset. Ancestry was assigned if the AAB individual was within 4 standard deviations of the mean for a given population group (European, South Asian, East Asian, African). All other individuals were assigned to “other” ancestry.

For the AAB European subset (*n* = 1,973), we calculated 20 PCs using GCTAv1.92 [31, 32], based on the genotyped SNPs, which were subsequently included as linear regression covariates in the PGS analyses. As input, we took *n* = 255,861 common genotyped SNPs with minor allele frequency > 0.05 in the Haplotype Reference Consortium dataset.

Familial relationships were inferred using pairwise identity-by-state estimation with the PLINK1.9—genome command. We used linkage disequilibrium-pruning (settings: window size 250 SNPs, step size 25 SNPs, VIF = 1.111 or equivalent to *r*^2^ = 0.1) to identify a set of 92,546 independent variants as input data. Relatedness checks were performed in two ways: 1) pairwise estimations across the entire dataset, and 2) pairwise estimations between self-reported family members. This enabled inference of parent-offspring (PI_HAT > 0.45, Z1 > 0.8, Z2 < 0.1), full-sibling (PI_HAT > 0.35 0.3 < Z1 < 0.8, Z2 > 0.1), monozygotic twins (PI_HAT > 0.8, Z2 > 0.8), and half-sibling (PI_HAT > 0.1, Z1 > 0.45, Z2 < 0.1) relationships, whereby PI_HAT refers to the estimated proportion of the genome that is inherited by descent (IBD) (P(IBD = 2) + 0.5*P(IBD = 1)), Z1 refers to P(IBD = 1) and Z2 refers to P(IBD = 2). These relationships were cross-referenced against the reported relationships in the AAB, and to match family members not otherwise linked by the ID system (e.g., where individuals within one family were recruited on different dates or assessment centres). For downstream analyses, we constructed a genetic relatedness matrix (GRM) from the SNP genotypes using GCTAv1.92 [31, 32].

To ensure consistency between child and parent genotypes within these family sets, we also performed a check for Mendelian errors within families using PLINK1.9 [27, 28], finding that the maximum parent–offspring Mendelian error rate was ~ 0.1%, consistent with the known error rate on Illumina SNP arrays, and implying no undetected sample mix-ups.

### Polygenic scoring

#### Description of input GWAS summary statistics

We used summary statistics from GWAS for height [33], ASD [4], IQ [34] and chronotype [35]. As an indicator of GWAS study power, these summary statistics had 2,380, 5,276, and 327 independent (*p* < 5e−8, *r*^2^ < 0.1) genome-wide significant associations, respectively, from analyses of *n* = 345,011, *n* = 46,350 (18,381 cases, 27,969 controls), *n* = 269,867 and *n* = 697,828 individuals.

#### Selection of UK Biobank (UKB) controls

Given that the UNR group had minimal exclusion criteria and was small (*n* = 117), UK Biobank (UKB) controls with European genetic ancestry were selected as an additional control group. These individuals were selected by projecting the *n* = 1,964 ASD participants of European ancestry onto the PCs derived using 137,102 genotyped SNPs from *n* = 436,227 UKB participants of European ancestry (GCTAv1.92,–project-loading command). For each ASD participant, the *n* = 5 UKB participants with the least Euclidean distance across 3 UKB principal components were taken as controls (Additional file 1: Fig. 1).

**Fig. 1:**
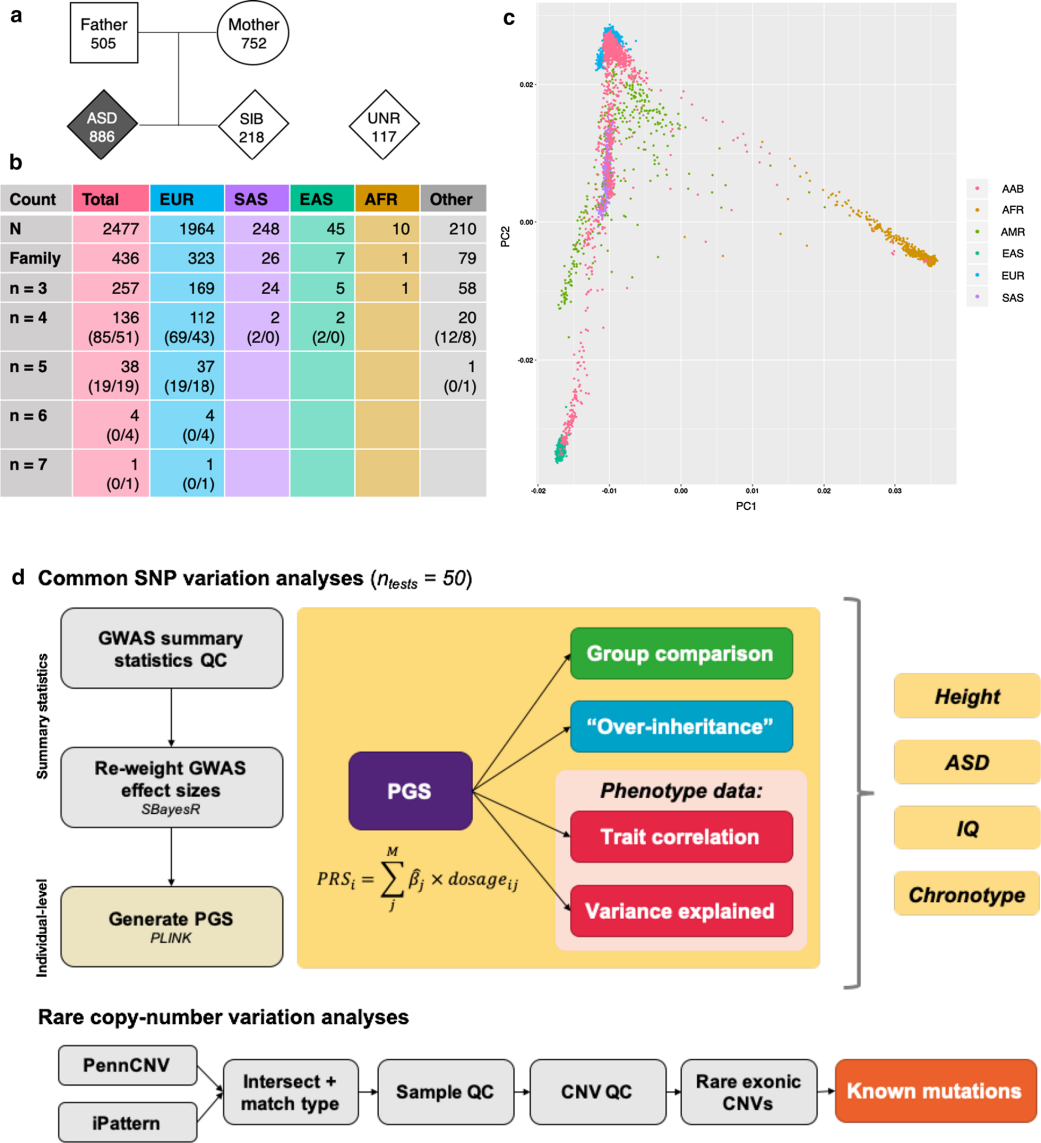
Characteristics of the AAB dataset. **a** Diagram showing cohort relationships. **b** Counts of familial relationships, split by ancestry. Ancestry is assigned to a family if the entire family is of the same genetic ancestry. N: total number of individuals in each ancestry group. Families: the number of families with both parents and at least one child. Subsequent rows (“*n* = ”) demonstrate the breakdown by family size (e.g., *n* = 3 denotes trios, *n* = 4 denotes quartets, and so on). The numbers in brackets refer to the number of families with one child on the autistic spectrum versus the number of families with multiple children on the autistic spectrum. c) Genetic ancestry of AAB individuals projected onto the first two principal components of the 1000 Genomes reference dataset. Pink denotes the AAB group. Acronyms for ancestry groups: AFR, African; AMR, American (Central and South) ; EAS, East Asian; EUR, European; SAS, South Asian. d) Overview of analyses performed, divided into common SNP analyses (upper) and rare copy-number variation analyses (lower). The number of tests used to calculate Bonferroni correction are also displayed

#### SBayesR PGS weighting

We generated polygenic scores (PGS) using SBayesR [33] – a Bayesian method that takes GWAS summary statistics as input. This method shrinks SNP effect sizes while still maximising variance explained by “binning” SNPs into a mixture of normally-distributed priors, accounting for linkage disequilibrium. SBayesR has been shown to outperform other PGS methods regardless of the underlying genetic architecture of the trait [33, 36]. SBayesR requires two inputs: 1) GWAS summary statistics from which HapMap3 SNPs with imputation INFO filter > 0.8 were extracted, retaining only those SNPs that passed QC in both AAB and UKB and 2) linkage disequilibrium matrices built using HapMap3 SNPs from a subset of 50,000 unrelated Europeans from the UKB. Additional file 2: Table 1 shows the number of SNPs used in each SBayesR analysis (intersection of HapMap3 SNPs across the GWAS discovery and AAB), and Additional file 2: Table 2 shows the SBayesR output. SBayesR was run with the default inputs: –pi 0.95, 0.02, 0.02, 0.01; gamma 0, 0.01, 0.1, 1; chain-length 10,000; burn-in 2000; out-freq 10, and using the –exclude-mhc flag. For height only, there was an additional step to filter GWAS SNPs with the software package DENTIST [37], to remove inconsistent imputed Z-scores based on the linkage disequilibrium reference matrix and observed GWAS Z-scores, which improved convergence of the SBayesR algorithm.

**Table 1:** Summary of CNV statistics between groups

Group	*n*_all	*n*_ind	*n* CNV	% with CNV	No. median	No. mean	ASD/ID CNV del	ASD/ID CNV dup	ASD/ID/DD genes del	ASD/ID/DD genes dup
ASD	885	263	330	29.72	0	0.37	11	4	5	3
FTR	504	134	167	26.59	0	0.33	0	0	1	0
MTR	752	232	280	30.85	0	0.37	3	0	3	0
SIB	218	57	68	26.15	0	0.31	0	0	0	0
UNR	116	37	40	31.90	0	0.34	0	0	0	0

n_all, number of individuals within the entire group; n_ind, number of individuals with a rare exonic CNV in the group; n_CNV, number of rare exonic CNVs in that group; % with CNV, % of individuals with a rare exonic CNV. no. median, median number of rare exonic CNVs; no. mean, mean number of rare exonic CNVs; ASD/ID CNV del, number of rare exonic deletion CNVs overlapping with the ClinGen + DECIPHER deletion CNV set; ASD/ID CNV dup, number of rare exonic duplication CNVs overlapping with the ClinGen + DECIPHER duplication CNV set; ASD/ID/DD genes del, number of rare exonic deletion CNVs overlapping with the Satterstrom et al. (2020) + DDD (2017) gene set; ASD/ID/DD genes dup, number of rare exonic duplication CNVs overlapping with the Satterstrom et al. (2020) + DDD (2017) gene set (100% overlap required)

**Table 2:** ASD/ID-associated CNVs detected in the AAB dataset

Diagnosed children							
Sample ID	Group	Sex	Type	CNV coordinates	ASD/ID-associated CNV	Reference coordinates	Other information
*CNVs with a critical gene*							
1101366	ASD	M	Dup	chr17:1,196,088–1,326,656	17p13.3 (Miller-Dieker syndrome) region (includes YWHAE and PAFAH1B1)^	chr17:1,247,833–2,588,909	Critical gene coordinates: YWHAE: chr17:1,247,569–1,268,350; PAFAH1B1: chr17:2,541,583–2,585,096
1101637	ASD	M	Dup	chr17:29,111,368–30,343,735	17q11.2 recurrent region (includes NF1)	chr17:29,097,069–30,264,027	Critical gene coordinates: chr17:29,422,328–29,701,173
*CNVs without a critical gene*							
4406214	ASD	F	Dup	chr15:22,321,690–32,515,100	15q11q13 recurrent (PWS/AS) region (BP1-BP3, Class 1)	chr15:22,832,519–28,379,874	100.00% overlap
3305166	ASD	M	Del	chr15:29,079,105–32,515,100	15q13.3 recurrent region (D-CHRNA7 to BP5) (includes CHRNA7 and OTUD7A)	chr15:32,019,621–32,445,405	100.00% overlap
1101486	ASD	M	Del	chr15:31,007,901–32,515,100	15q13.3 recurrent region (D-CHRNA7 to BP5) (includes CHRNA7 and OTUD7A)	chr15:32,019,621–32,445,405	100.00% overlap
1101365	ASD	M	Del	chr15:31,115,226–32,515,100	15q13.3 recurrent region (D-CHRNA7 to BP5) (includes CHRNA7 and OTUD7A)	chr15:32,019,621–32,445,405	100.00% overlap
4406241*	ASD	M	Del	chr16:21,973,913–22,414,463	Recurrent 16p12.1 microdeletion (neurodevelopmental susceptibility locus)	chr16:21,946,524–22,467,284	84.60% overlap
3305177	ASD	M	Del	chr16:28,832,565–29,044,745	16p11.2 recurrent region (distal, BP2-BP3) (includes SH2B1)	chr16:28,822,635–29,046,499	94.78% overlap
1101491	ASD	F	Del	chr22:18,877,787–21,461,607	22q11.2 recurrent (DGS/VCFS) region (proximal, A-D) (includes TBX1)	chr22:15,912,231–21,465,672	99.84% overlap
4406202	ASD	F	Del	chrX:6,488,784–8,135,053	Xp22.31 recurrent region (includes STS)	chrX:6,455,812–8,133,195	98.02% overlap
*Parents*							
4411283*	MTR	M	Del	chr16:21,956,457–22,414,463	Recurrent 16p12.1 microdeletion (neurodevelopmental susceptibility locus)	chr16:21,946,524–22,467,284	87.95
2215012	MTR	F	Del	chr22:19,036,154–20,244,259	22q11.2 recurrent (DGS/VCFS) region (proximal, A-B) (includes TBX1)	chr22:18,912,231–20,287,208	87.86
1116571	MTR	F	Del	chrX:6,456,940–8,135,053	Xp22.31 recurrent region (includes STS)	chrX:6,455,812–8,133,195	99.93

ASD/ID-associated CNVs were taken from ClinGen [47] and DECIPHER [48] datasets, filtering for ASD/ID-associated loci. For reference CNVs with a critical gene, AAB CNVs were annotated where there was any overlap with the critical gene, with the critical gene coordinates provided in the "Other information" column. For reference CNVs without a critical gene, the AAB CNV was called as overlapping with an ASD/ID-associated CNV based on ≥ 80% overlap with the reference coordinates, with percentage overlap provided in the "Other information" column. Genome coordinates are hg19. Biobank Sample IDs have been anonymized

*Refers to parent–child pairs between which ASD/ID-associated CNVs appeared to be inherited in this dataset

^Note that the CNV for ID 1101366 only overlaps the YWHAE critical gene

#### PGS calculation

To generate PGS for each trait, we multiplied the best guess genotypes in the target sample (i.e. AAB individuals and UKB controls) by the effect sizes (reweighted by SBayesR [33], with the addition of DENTIST [37] for the height analysis), using the PLINK –score function. We restricted analyses of the target dataset to the subset of participants of inferred European ancestry. The PGS scores were standardised by subtracting the mean and dividing by the standard deviation of the UKB controls. We generated PGS from four sets of GWAS summary statistics: height from the UKB [33], excluding the controls that we had selected, ASD [4], IQ [34] and chronotype [35]. Height was selected as a benchmarking phenotype that has large GWAS discovery sample size and so is well-powered for PGS. ASD is of course directly relevant to the AAB cohort, while IQ and chronotype are genetically-correlated with ASD [4]. Given our small sample size, we eschewed analysis of additional traits to avoid incurring a high multiple testing burden.

#### Between-group PGS differences

We tested for a mean difference in PGS for each trait between ASD, SIB and UNR AAB experimental groups using Z-tests. To improve power to test for differences, we added the group of unrelated controls of European ancestry from the UKB (as described above).

#### Over-transmission of common genetic variation for ASD

We tested for over-transmission of common genetic variation for ASD from parents to their children with and without autism. For *n* = 330 individuals diagnosed with ASD and *n* = 145 undiagnosed siblings with parental genotyping data, we tested for polygenic transmission disequilibrium using the pTDT software [38]. Briefly, this tests for a deviation of the child’s PGS from the mid-parental PGS (which represents the null). We also looked for evidence of assortative mating for ASD only. For this, we tested for correlation of PGS between the parental pairs within the family data.

#### Relationships between PGS and phenotypes

We calculated correlations between PGS of multiple traits and various phenotypes of interest recorded within the AAB. We calculated the proportion of variance in standardised phenotype residuals explained by PGS, where phenotype residuals resulted from regression on covariates that included age, sex and 20 PCs (calculated using GCTA on *n* = 255,861 common genotyped SNPs with minor allele frequency > 0.05 in the Haplotype Reference Consortium dataset, and only in the European subset of *n* = 1,973 for whom we generated PGS), which were selected for inclusion based on backwards stepwise regression.

#### Multiple testing correction

We used the Bonferroni method to correct for multiple testing across all PGS analyses (50 tests, *p* ≤ 0.05/50 or *p* ≤ 1e-3). Phenotype-PGS associations that were tested for both variance and correlation were counted as one test, as these statistics are mathematically related. With this threshold, we had ~ 80% power to detect a difference of 0.17 SDs in PGS mean when comparing the ASD (*n* = 697) and UKB groups (*n* = 3,490). For the PGS prediction analyses of quantitative traits, we had ~ 80% power to detect *R*^2^ = 0.014 in a linear regression with 1 predictor, alpha = 1.0e−3 (after multiple testing) and sample size of all 1,222 children (noting that sample size was restricted to the available phenotype data).

### Copy number variant (CNV) calling

We followed the Psychiatric Genomics Consortium CNV analysis pipeline [39], with a few modifications. CNVs were identified in each AAB individual using consensus calling from PennCNV and iPattern. PennCNV [13] takes as input signal intensity data from SNP genotyping arrays (Log R Ratio and genotypes), and uses a hidden Markov model-based approach to achieve kilobase resolution of CNVs [13]. In contrast, iPattern [10] takes the intrachip-normalised X and Y values as signal intensity measures and performs joint analysis across multiple individuals to identify CNVs.

We used the Bioconductor GenomicRanges package [40] to process and perform operations on genetic coordinate data. Different CNV calling software may break up large CNVs and compromise consensus calling; to account for this we merged CNV calls from PennCNV and iPattern separately using a greedy algorithm:

1. Individuals with > 5 CNVs on one chromosome were identified;
2. For that individual, CNVs were split into type (gain or loss), as an artificially-split CNV should have concordant type across its segments;
3. Adjacent CNVs with gaps ≤ 100 kb were merged in a greedy algorithm;
4. Adjacent CNVs with gaps ≤ 25% of the length of the neighbouring CNV were merged in a greedy algorithm.

We obtained consensus CNV calls for each individual by intersecting CNV calls from both methods and retaining those with ≥ 50% overlap, ensuring that copy number (gain or loss) was matching in each method. A total of 10,752 consensus CNVs were identified using this approach.

Sample QC was performed using summary statistics from the PennCNV output. We removed *n* = 137 samples where any of the LRRSD (Log R Ratio), BAFSD (B Allele Frequency Standard Deviation) or GCWF (GC-content Wave Form) statistics (based on autosomal data) had a value more than three standard deviations from the mean. We also applied a filter for samples where CNVs made up greater than 20% of any chromosome to exclude aneuploidy. This step identified *n* = 2 individuals – one participant from the ASD group with known Down syndrome (trisomy 21), and one participant with diagnosed Smith-Magenis syndrome whom had been included in the UNR group. CNVs were excluded if:

1. the PennCNV confidence score was < 10;
2. there was any overlap with centromeres or the major histocompatibility complex region (hg19 coordinates chr6:28,477,797–33,448,354), the latter due to the complexity of this region;
3. there was > 50% overlap with a segmental duplication;
4. the CNV was common. This was determined by firstly identifying genomic regions that were overlapped by 25 or more CNVs (a threshold of 25 was chosen as this corresponds to ~ 1% of the AAB population). CNVs with ≥ 50% overlap with these regions were considered “common” and therefore excluded;
5. the CNV was < 10 SNPs in length and/or < 20 kb in length;
6. the CNV was common or benign: defined as ≥ 70% overlap with CNVs classified as benign by Zarrei et al*.* [41] using their “inclusive” criteria; considered as common in the DECIPHER study[42]; or maximum frequency across populations ≥ 1% in the gnomAD v2 dataset [43, 44]; and
7. the CNV overlapped exclusively with non-coding regions, as determined using RefSeq hg19 gene annotation coordinates[45]; and
8. the CNV was non-genic.

After QC, we excluded one additional individual for whom 21 CNVs had been called (the next-highest number of CNVs for an individual was four), leaving 885 CNVs from 723 individuals remaining for subsequent analysis.

### CNV annotation

We annotated CNVs using hg19 cytoband and gene coordinates imported from biomaRt [46]. We defined cytoband overlap when ≥ 50% of the called CNV length lay within the cytoband. Gene overlap was defined as any overlap with coding exons. We checked for overlap between CNVs in the AAB dataset and a total of 51 pathogenic CNV regions involved in neurodevelopmental conditions selected from clinical databases (ClinGen [47] and DECIPHER [48, 49]) for being associated with ASD and ID. To define overlap, we divided pathogenic ASD/ID CNVs into those with and without a critical gene. CNVs with a critical gene required overlap with any exon, whereas CNVs without a critical gene required ≥ 80% of the pathogenic CNV region. We also looked for overlap of the AAB CNVs with the 102 genes identified by the largest whole exome sequencing study of ASD to date [12], and 93 genes associated with developmental disorders from the Deciphering Development Disorders (DDD) study [50] – a total of 158 unique genes.

## Results

### Characteristics of the Australian Autism Biobank (AAB)

A summary of familial relationships and genetic ancestry in the AAB is provided in Fig. 1. AAB participants passing genotyping QC (*n* = 2,477 individuals, *n* = 546 family groupings, *n* = 436 families with both parents and at least one affected child) were predominantly of European ancestry (*n* = 1,964 individuals, *n* = 323 families, *n* = 154 families with more than one child in the AAB), with representation from other populations including South Asian (*n* = 248 individuals, *n* = 26 families), East Asian (*n* = 45 individuals, *n* = 7 families) and African (*n* = 10 individuals, *n* = 1 family). All other individuals of admixed ancestry were classified into an “Other” group (*n* = 211 individuals, *n* = 79 families, *n* = 21 families with more than one child in the AAB) (Fig. 1b). For families with multiple participating children (i.e., total family *n* > 3), Fig. 1b also summarises the number of families with one child on the spectrum versus the number of families with multiple autistic children.

### Polygenic risk scoring

#### Between-group PGS comparisons

**Fig. 2:**
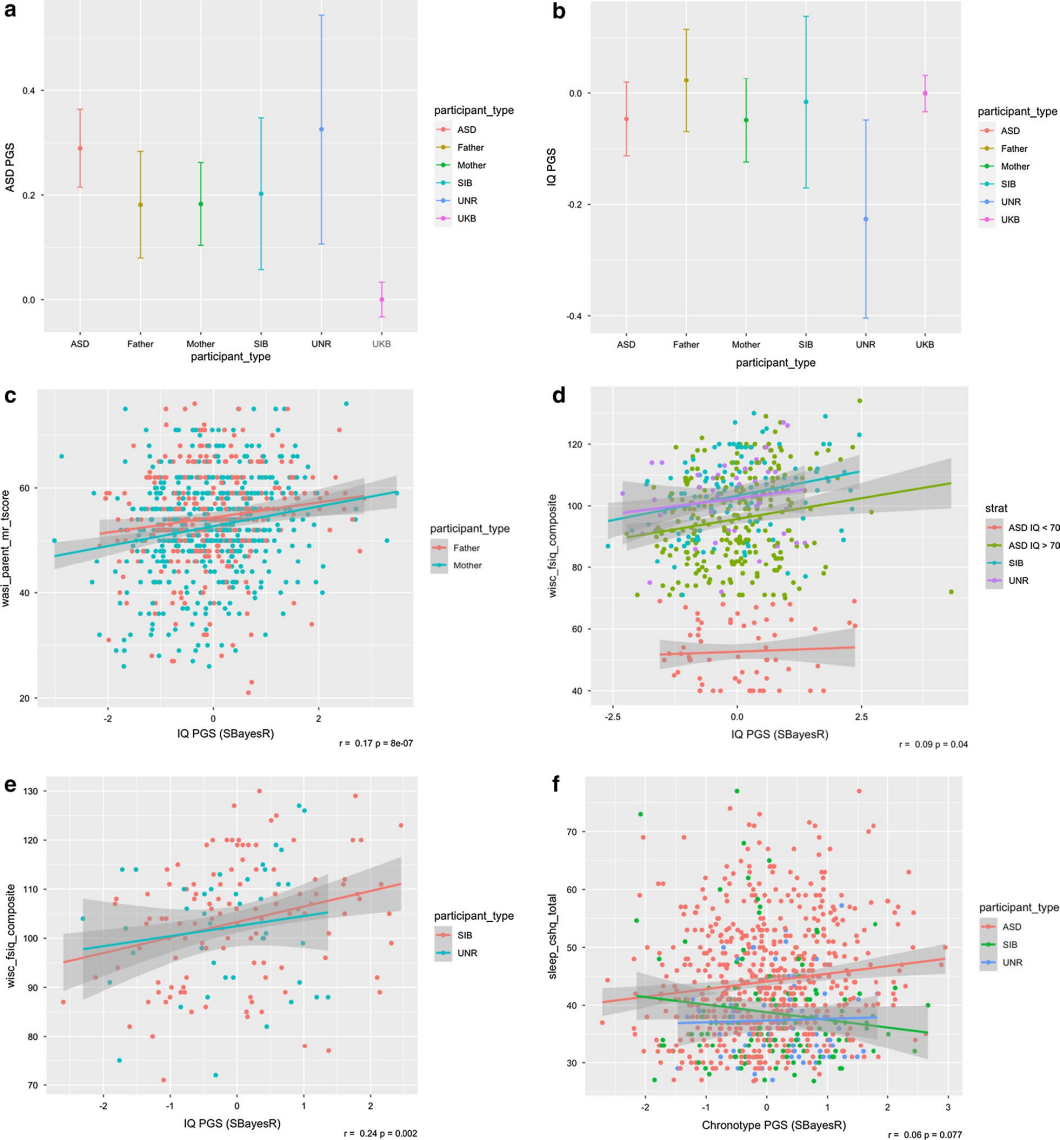
PGS results: Mean PGS ± 95% CI for the ASD, SIB and UNR groups for **a** ASD, and **b** IQ (Additional file 2: Table 3). Scatterplots illustrating correlation between the following pairs of traits, with coefficient and *p*-value in the bottom right corner of each panel for the single combined analysis: **c** IQ PGS and measured IQ (WASI) in parents with fathers in red and mothers in green (overall *r* = 0.17, 8.0e−7); **d** IQ PGS and measured IQ (WISC fsiq composite) in the AAB ASD group with IQ < 70 (red), ASD group with IQ ≥ 70 (green), SIB group (blue) and UNR group (purple), (overall *r* = 0.1, *p* = 4.0e−2); **e** IQ PGS and measured IQ (WISC fsiq composite) in the SIB (red) and UNR (green) groups (overall *r* = 0.24, *p* = 2.1e−3); (f) chronotype PGS and Children’s Sleep Habits Questionnaire in the ASD (red), SIB (green) and UNR (blue) groups (overall *r* = 0.06, *p* = 7.7e−2). The correlation coefficient for the ASD subset in red is *r* = 0.13, *p* = 1.9e−3)

Using the PGS from each AAB participant of European ancestry, we first tested whether there were group differences in genetic propensity for various traits. As a negative control, we found no differences in mean height PGS (Additional file 1: Fig. 2, Additional file 2: Table 3). We next tested autism-associated traits. The mean of the ASD PGS was higher in the ASD group (*p* = 6.1e−13) than the UKB group after multiple-testing correction (Fig. 2) (Additional file 2: Table 3). The SIB (*p* = 4.9e−3) and UNR (*p* = 3.0e−3) groups also had higher mean ASD PGS than the UKB at a nominal threshold (Fig. 2) (Additional file 2: Table 3). There was no evidence for a significant difference in mean ASD PGS between the ASD and SIB groups in the AAB, or between ASD and UNR controls (Fig. 2a). IQ is of interest in relation to ASD as ID commonly co-occurs, and yet there is positive genetic correlation based on common SNPs. For IQ PGS, we observed no difference between groups in the AAB, nor with the UKB controls, after multiple testing correction (Fig. 2b). We investigated chronotype as sleep disturbances also commonly co-occur with autism [51]. We did not find differences in chronotype PGS between the ASD, SIB and UNR groups in the AAB (Additional file 1: Fig. 2, Additional file 2: Table 3). For chronotype, we did not test for differences between AAB groups and the UKB controls, as the input GWAS also included UKB participants.

**Table 3:** List of genes from the Satterstrom et al. (2020) whole-exome sequencing study [12] and DDD study (2017) [50], overlapping with CNVs in the AAB dataset

Sample ID	Group	Sex	CNV coordinates	Gene	Description	Overlap % of gene	Cytoband
Diagnosed children							
Deletions							
1101306*	ASD	F	chr2:148,730,454–148,883,419	MBD5	methyl-CpG binding domain protein 5	21	2q23.1
1101211+	ASD	M	chr10:27,978,030–28,041,669	MKX	mohawk homeobox	78	10p12.1
3305052	ASD	M	chr19:10,609,319–12,464,434	ELAVL3	ELAV like neuron-specific RNA binding protein 3	100	19p13.2
4406296^	ASD	M	chr20:61,824,507–62,321,517	KCNQ2	potassium voltage-gated channel, KQT-like subfamily, member 2	100	20q13.33
4406297^	ASD	M	chr20:61,802,599–62,268,955	KCsNQ2	potassium voltage-gated channel, KQT-like subfamily, member 2	100	20q13.33
1101417	ASD	M	chr2:32,277,654–32,818,823	SPAST	spastin	100	2p22.3
1101240	ASD	M	chr4:6,104,865–7,415,038	KIAA0232	KIAA0232	100	4p16.1
4406214	ASD	F	chr15:22,321,690–32,515,100	GABRB3	gamma-aminobutyric acid (GABA) A receptor, beta 3	100	15q12
Parents transmitting CNVs							
1116306*	Mother	F	chr2:148,730,454–148,883,419	MBD5	methyl-CpG binding domain protein 5	21	2q23.1
1116211+	Mother	F	chr10:27,978,030–28,041,669	MKX	mohawk homeobox	78	10p12.1
4411296^	Mother	F	chr20:61,802,599–62,321,517	KCNQ2	potassium voltage-gated channel, KQT-like subfamily, member 2	100	20q13.33

Genes associated with ASD, ID and DD were taken from the Satterstrom et al. whole-exome sequencing study [12], and the DDD gene set [50]. If the AAB CNV was a deletion, gene overlap was called if there was any exonic overlap; if the CNV was a duplication, gene overlap was called if there was full overlap. A full list of CNVs overlapping ASD/ID/DD-associated genes – including those from parents that were not transmitted to children in the dataset – is provided in Supplementary Table 8d

*, ^+^ ,^ denote CNVs shared by individuals within the same family (either inherited from parents, or shared between siblings), suggesting inheritance. CNV, copy number variant

#### Over-transmission of genetic variation for ASD

The pTDT analysis provided no evidence for difference in ASD or IQ PGS between the ASD and SIB groups (Additional file 2: Table 4). We did not find evidence of assortative mating between parental pairs on the basis of ASD PGS (*n* = 324 European pairs, *r* = 0.06, *p* = 0.28), nor in the analysis of height PGS (*r* = 0.09, *p* = 0.11), likely due to the small size of this subset.

#### Prediction of autism and related phenotypes

The deep phenotypic data included in the AAB allowed us to leverage publicly-available GWAS summary statistics to perform prediction analyses into our dataset.

*Height*: Height is considered a model genetic trait because it is highly heritable and summary statistics from very large GWAS are available. We used height as a control trait, and to validate our analysis pipeline, because it is not expected to exhibit differences between the participant groups (although a higher proportion of participants in the ASD than SIB groups would be expected to carry major de novo CNVs associated with reduced stature [52]). We considered adults and children separately, as adults have stable height, and should be predicted more accurately. In the subset of AAB adults, height was strongly correlated with height PGS (*r* = 0.37, *p* = 1.0e−30; correlation on residuals after adjusting for age and sex: *r* = 0.52, *p* = 2.7e−66), and the PGS accounted for 27% of the variance after adjusting for covariates (Additional file 2: Tables 5, 6). In comparison, height PGS in AAB children captured only 13% of the variance (correlation: *r* = 0.14, *p* = 9.2e−11), presumably because of age effects (Additional file 2: Table 6). As expected, there was no evidence for between-group differences in height PGS or for over-transmission of common genetic variation for height in the ASD group compared to the SIB group (Additional file 2: Tables 3, 4). Thus, the height analysis performed as expected, justifying the use of the UKB control group and is consistent with no residual genetic stratification between the AAB groups and the UKB controls.

*ASD*: We determined whether the ASD PGS based on Grove et al*.* [4] predicted diagnosis of ASD in the AAB, in addition to other ASD-related phenotypes, including ADOS-2 calibrated severity score within the ASD group, Social Responsiveness Scale t-score in the SIB and UNR groups, and the Communication Checklist-Adult in parents. These tests were chosen as there were no autism spectrum questionnaires that were available across all participant groups. We also tested for association between ASD PGS and indicators for age of onset (age of first parental concern and age of diagnosis) and other ASD-associated phenotypes, including Short Sensory Profile-2 raw score (only available in the ASD group), WISC-IV composite score (or MSEL non-verbal developmental quotient) in children and WASI matrix reasoning score in parents. For cognitive ability phenotypes, we also stratified children into ASD and SIB/UNR groups, as we hypothesised that—given positive genetic correlation with autism [4]—the ASD PGS may have different relationships with IQ or developmental quotient depending on diagnostic status. No associations (passing Bonferroni correction) were found between ASD PGS and ASD diagnostic status, likely because the ASD GWAS is underpowered. However, there were nominal associations between ASD PGS and quantitative traits such as MSEL non-verbal developmental quotient (*r* = -0.11, *p* = 7.4e−3 in the ASD group alone) and marginal association with parental WASI matrix reasoning score (*r* = 0.07, *p* = 5.5e−2) (Additional File 2: Tables 5, 6).

*IQ*: IQ is a trait of interest as others have reported positive genetic correlation with autism [4], which is paradoxical as ID commonly co-occurs with autism. Hypothesising that diagnostic status may affect the relationship, we considered ASD and SIB/UNR groups separately. IQ PGS showed a significant positive correlation with parental IQ, as measured by the WASI matrix reasoning domain (*r* = 0.17, *p* = 8.0e−7) (Fig. 2c, Additional File 2: Table 5), and captured 4% of the variance of the residuals of the baseline model (model *p* = 2.85e−9) (Additional File 2: Table 6). There was a nominally-significant correlation between IQ PGS and WISC-IV composite score (Fig. 2d-e, Additional File 2: Table 5) in the SIB/UNR group (*r* = 0.24, *p* = 2.1e−3) (Fig. 2e), and within this group, the IQ PGS explained 6.7% of variance (*p* = 6.0e−4, an additional 213% compared to 3% from the baseline model) and made a significant contribution to the model (beta = 3.21, *p* = 6.0e−4) (Additional File 2: Table 6). In contrast, there was no evidence for a significant correlation in the ASD group (*r* = 0.07, *p* = 0.24) (Fig. 2d, Additional File 1: Fig. 5, Additional File 2: Table 5), including when the ASD group was stratified by ID (Additional File 1: Fig. 5b). In the corresponding variance analysis within the ASD group, the PGS captured 0.4% of variance in addition to the baseline model, which was not significant (*p* = 0.12) (Additional File 2: Table 6). There were no significant relationships between IQ PGS and MSEL non-verbal developmental quotient, age of diagnosis, age of parental concern, and Short Sensory Profile-2 raw score (Additional File 2: Table 5).

To screen for ascertainment bias in relation to overlap between ASD and IQ, we looked for relationships between mid-parental matrix reasoning IQ score and both age of diagnosis and ID in their children. There was no correlation between age of diagnosis and mid-parental matrix reasoning IQ, but we note that the distribution had a slight right-shift in distribution in mid-parental matrix reasoning IQ in trios where the child did not have ID (defined as WISC-IV IQ comparison score ≥ 70)  (Additional File 1: Fig. 6).

*Chronotype*: Sleep issues are common among people on the spectrum [53, 54], so we investigated whether common genetic variants contribute to propensity for sleep conditions. There was no evidence for a significant correlation between chronotype PGS and CSHQ raw score among all children (*r* = 0.06, p = 7.7e−2), but we observed a nominally-significant positive correlation (*r* = 0.13, *p* = 1.9e−3) in the ASD group (Fig. 2f, Additional File 1: Fig. 7, Additional File 2: Table 5). Within the ASD group, the chronotype PGS explained 1% of the variance (*p* = 3.4e−3) in CSHQ score, a 34% increase compared to the baseline model, which had adjusted R^2^ = 4% (Additional File 2: Table 6, Additional File 1: Fig. 7).

### Copy number variants (CNVs)

We obtained consensus calls for 885 CNVs from 723 individuals in the AAB cohort after QC (summarised in Table 1). As a first step, we determined whether our pipeline validated previously-identified pathogenic CNVs. Seven out of eight clinical genetic diagnoses (Additional file 2: Table 8a) were identified by both PennCNV and iPattern, with the exception of an individual with Phelan-McDermid syndrome (caused by a 6,635 bp 22q13 deletion of chr22: 51,159,408–51,166,043), which was detected by PennCNV, but not by iPattern. Of the seven remaining, one child with Smith-Magenis syndrome whom had been assigned to the UNR group was removed from subsequent analyses; two had CNVs that did not survive the QC filters (one overlapped with a contig region with poor-quality mapping, whereas another was purely intronic); and two events were not in the ClinGen + DECIPHER CNV set (Additional file 2: Table 8a). We also note that the genotyping QC process identified one child in the ASD group with Turner syndrome (XO) and one child in the UNR group with Klinefelter syndrome (XXY).

#### CNV annotation

We identified 13 individuals with CNVs that overlapped high-confidence ASD/ID-associated CNVs from the ClinGen [47] and DECIPHER [48, 49] databases (Table 1, Additional file 2: Table 7a, b). Four of these CNVs had been reported in the AAB phenotype dataset (a total of eight had been reported, Additional file 2: Table 8a), although it is important to note that there was no formal field for genetic diagnosis, and those that were recorded were offered by parents in response to the child’s medical history. Ten of these were from the ASD group, and three from their mothers. The phenotypic data of the participants with overlapping ASD/ID-associated CNVs was closely inspected, finding that many of these participants had reported one or more clinical features that were consistent with the genetic diagnoses, including DD, ID (measured using WISC-IV or diagnosed previously), history of seizures, macrocephaly and/or sleep disturbances (Additional file 2: Table 8b). We also identified large CNVs that were > 1 Mb in length, among participants whom did not have an ASD/ID-associated CNV. There were 37 individuals whom met these criteria (19 ASD, 1 SIB, 3 UNR, 11 mothers, 3 fathers), including some that were inherited, or demonstrated familial patterns: a 1.3 Mb 16p23.1 deletion occurring in a father-child (ASD) pair, a 1.9 Mb 2q37.3 deletion occurring in a mother–child (ASD) pair, a 2.4 Mb 4q35.2 duplication occurring in a mother and two of her children (both in the UNR group), and an identical twin pair (both in the ASD group) sharing a 2.3 Mb 1p34.2 deletion. The genetic coordinates, corresponding cytobands and phenotypic information for these individuals are provided in Additional file 2: Table 8c.

A further 12 individuals (Tables 2, 3, Additional file 2: Table 8d) were found to carry CNVs overlapping with coding sequences of genes in either the list of ASD-associated genes identified in the most recent ASD whole-exome sequencing study [12] or the list of genes associated with DD reported by the DDD study [50] (Additional file 2: Table 7b). Eight of these CNVs were identified in the ASD group and four were present in parents (three of which appeared to be transmitted to children, and are displayed in Table 3; CNV information for the additional parent is provided in Additional file 2: Table 8d).

**Fig. 3:**
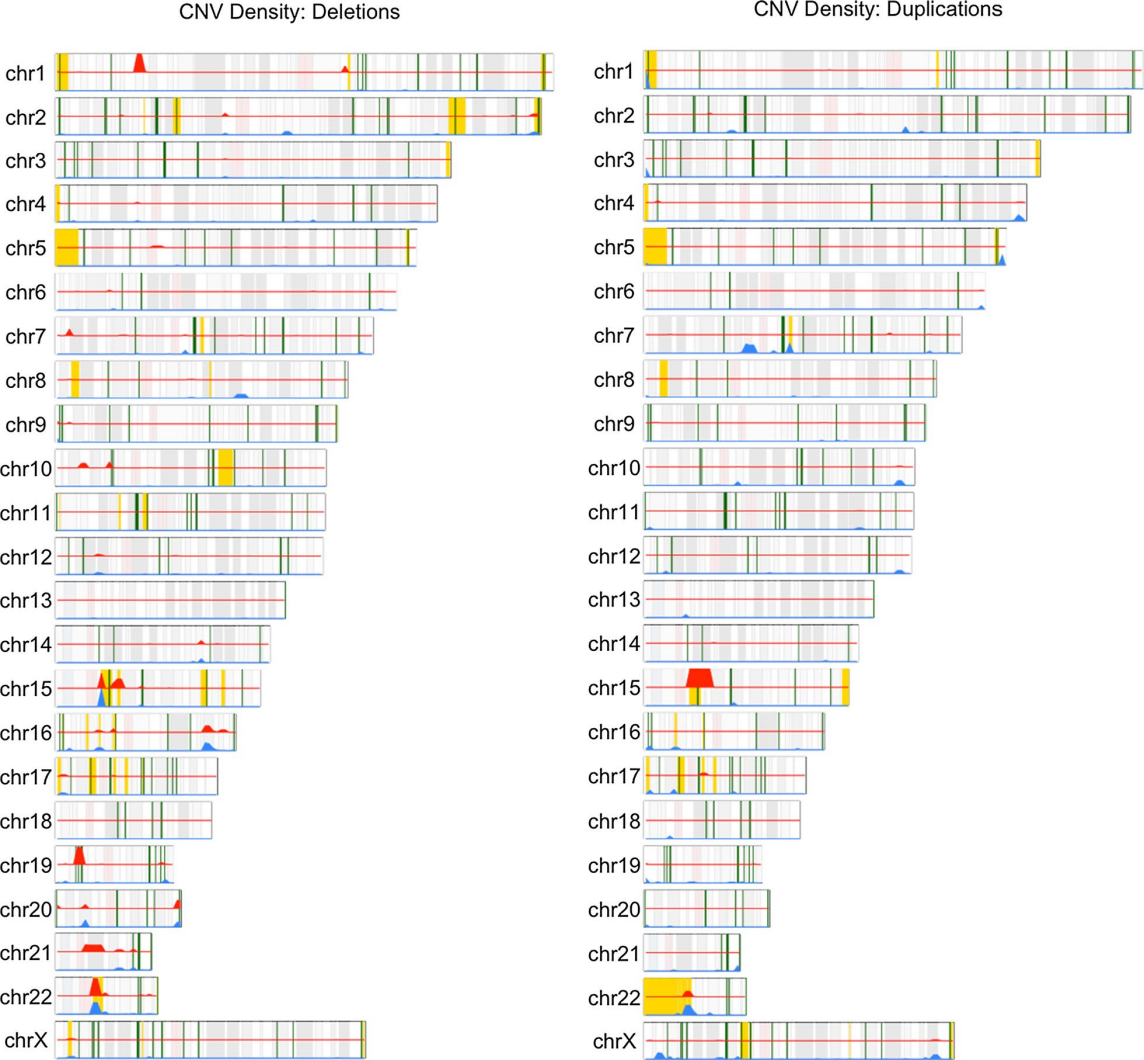
Karyograms showing location and density of deletion and duplication CNVs identified in the AAB cohort. Red density track represents CNVs detected in the ASD subset. Blue density track represents CNVs detected in the non-ASD subset (undiagnosed siblings, unrelated undiagnosed children, and parents), noting that there are instances in which parents have CNVs overlapping ASD/ID-associated regions. Yellow regions depict ASD/ID-associated CNVs from ClinGen [47] and DECIPHER [48, 49] (Additional file 2: Table 7a). Green regions denote ASD/ID/DD-associated genes reported by Satterstrom et al. [12] and DDD [50] (Additional file 2: Table 7b). Karyogram generated using the karyoploteR package [55]

A density plot of CNV regions (separated into deletions and duplications from the ASD/ID-associated CNV dataset) across all individuals, in relation to chromosome, cytoband, ASD/ID-associated CNVs and ASD/ID/DD -associated genes is provided in Fig. 3.

## Discussion

We report on polygenic variation and rare ASD/ID-associated CNVs within the AAB dataset [14]. We first characterised the genetic ancestry and family relationships of participants (Fig. 1). We next performed analyses of ASD PGS, finding that the ASD group had significantly higher PGS than UKB controls after multiple-testing correction. There were no significant relationships between the ASD PGS and many phenotypes relating to autism and related traits, likely because the ASD GWAS is still relatively underpowered; we anticipate that future releases of larger GWAS meta-analyses from the Psychiatric Genomics Consortium should improve prediction. In contrast to the ASD phenotype, we identified PGS-phenotype associations for IQ in the SIB/UNR groups and among parents that survived multiple testing correction, as well as nominal associations between chronotype and CSHQ score in the ASD group in both the correlation and variance analyses. This result is expected, as the contributing GWAS summary statistics are better-powered. From a rare variation standpoint, we also called CNVs, and found overlap with high-confidence ASD/ID-associated CNVs and genes in a subset of AAB participants. A major strength of this dataset is the breadth of its phenotyping and biological sample collection, which facilitates integration with other autism-focused datasets such as the Simons Simplex Collection (SSC) and Simons Foundation Powering Autism Research for Knowledge (SPARK); a summary of overlapping phenotypes is provided in Additional file 2: Table 9.

We found that the ASD group had significantly higher ASD PGS scores compared to UKB-selected controls that passed multiple-testing correction (*p* = 6.6e−13); however, there were no significant differences between the AAB groups. This may be explained by the small size of these groups in relation to the UKB control group, or alternatively, it may reflect increased ASD risk within all AAB groups compared to the population, which is possible given the recruitment setting and lack of exclusion criteria. We also found nominal evidence of over-transmission of ASD risk in the pTDT analysis (*p* = 5.8e−2), consistent with observations of over-transmission of common genetic risk in families with a child diagnosed with ASD [38]. These weak associations likely reflect small sample size within the AAB, and insufficient power in the discovery ASD GWAS [4]. To confirm that PGS differences between the AAB groups and UKB controls were not a reflection of residual population stratification, we compared PGS for height between AAB groups and UKB controls and found no differences.

There is a paradoxical relationship between genetic risk for autism and IQ, insomuch as a positive genetic correlation exists between autism and IQ based on common SNPs [4], but ASD frequently co-occurs with ID. Hence, we investigated the genetic relationship between IQ and autism in the AAB. We found no evidence for a relationship between IQ PGS and measured IQ in the ASD subset with ID (defined as IQ < 70) (Fig. 2d, Additional file 1: Fig. 5b), consistent with the prevailing view that this co-occurring condition reflects overlapping rare genetic variation, rather than the common variation captured by PGS [56]. This was also supported by the finding that there were no statistically-significant differences in ASD and IQ PGS between the ASD and SIB groups.

Sleep disturbances are commonly comorbid with autism [51], and the most recent ASD GWAS identified a positive genetic correlation with chronotype [4]. Consistent with this, we found a nominally-significant positive correlation (*r* = 0.13, *p* = 1.9e−3) between chronotype PGS and CSHQ raw score in the ASD group, as well as a nominally-significant contribution to the baseline model (% change = 34.4%, increase in *R*^2^ from the PGS = 0.01, *p* = 3.4e−3) (Additional file 1: Fig. 7). These associations were not evident in the SIB and UNR groups (Fig. 2, Additional file 1: Fig. 7). In the chronotype GWAS, the phenotype was scored from − 2 to 2, as a spectrum from “definitely evening” to “definitely morning” people, respectively. Hence, the results here suggest that sleep disorders among the ASD group are associated with common genetic variation for greater “morningness”. We note that the CSHQ raw score captures a wide variety of sleep disturbances, in addition to differences in chronotype. However, when we generated a composite core of questionnaire items that were more directly relevant to chronotype, there was no significant correlation with chronotype PGS (Additional file 2: Table 5). This may suggest that variants associated with chronotype magnify broad sleep disturbances in the ASD group.

We also looked for evidence of assortative mating by correlating ASD PGS scores in spouse-pairs (Additional file 1: Fig. 4), but did not find any significant correlation (*r* = 0.06, *p* = 0.28, *n* pairs = 324). We also performed this analysis for height, again finding no significant evidence for assortative mating (*r* = 0.09, *p* = 0.11, *n* pairs = 322). In both analyses, the point estimates are consistent with reported assortative mating correlations [57, 58], and our sample size contributes to the non-significance of the estimates.

For both height and IQ, PGS was a superior predictor in parents compared to children (Additional file 1: Fig. 3, Additional file 2: Tables 5, 6). This may be related to greater “stability” in measured phenotypes in adults (e.g., negligible effect of age in height among adults, compared to variability in child growth rate), and also because these GWAS were conducted in adults.

We note that iPattern failed to identify one individual whom had been diagnosed with Phelan-McDermid syndrome (caused by a 22q13 deletion), whereas this was identified by PennCNV (called CNV length = 6.6 kb, which is small for 22q13 deletions, which are usually 142 kb in size). This reflects the increased sensitivity of PennCNV, as CNVs are called on an individual basis, whereas iPattern calls CNVs within batches.

## Limitations

Our study has a number of limitations. Firstly, autism is highly heterogeneous, both genetically and clinically, necessitating large sample sizes for discovery. Hence, we downplay the risk of the identified CNVs that are not already known to overlap high-confidence ASD-associated CNVs. Insufficient power is also an issue for the ASD GWAS summary statistics that were used to calculate PGS. The availability of better-powered GWAS in the near future should improve the power of the prediction analyses performed here. We hope that this dataset will contribute to future meta-analyses. Furthermore, our sample is relatively small, particularly in relation to the SIB and UNR groups, and the results require replication in independent datasets to be generalisable.

We note that the UNR group had higher mean ASD PGS and lower mean IQ PGS than might be expected in a community sample (Fig. 2a,b). However, this group was comparatively small (*n* = 117), and the standard errors are large, so it is difficult to draw further inference from these results. The majority of UNR participants were recruited from community settings, but some were contacted through association with health care providers and there were minimal exclusion criteria (ie., no diagnosis of ASD), which could contribute to the observed results.

In our PGS analyses, we remained cognisant of multiple-testing issues. We selected the statistical tests we performed in a hypothesis-driven manner and applied a Bonferroni correction across all *p* values calculated in this analysis. However, there are other interesting PGS-phenotype relationships that may be interrogated in the AAB that we opted not to test, given the power of the current datasets available to us.

We used genetic PCs as covariates in our analysis, and also to select UKB controls. Like many genetic analyses of this nature, we acknowledge that it is difficult to balance the competing issues of residual population stratification with the potential for over-correction. To address the former, we matched UKB controls within 2 standard deviations across 3 UKB European PCs (the maximum possible to achieve matching of *n* = 5 UKB individuals per AAB participant), and included 20 PCs from the AAB European cohort in the regression models. To account for the latter, we used a backwards stepwise model to only select the most salient genetic PCs as covariates. Our PGS analyses for height provided support that the AAB and UKB samples were genetically well-matched.

We note that our ability to call CNVs using SNP genotyping data is limited in resolution and sensitivity. Whole-genome sequencing is the gold-standard; however, this data has not yet been generated in the AAB cohort.

## Conclusions

Here, we characterise common genetic variation and rare ASD/ID-associated CNVs in the AAB cohort using SNP genotyping and demonstrate the utility of leveraging publicly-available data to predict traits within the AAB’s accompanying deep phenotypic dataset. We demonstrate that PGS for IQ, chronotype and height predict these phenotypes within the AAB dataset, and we identify individuals carrying ASD and ID-associated CNVs. Although this sample is not powered for discovery, these results will be important to integrate into future analyses of other omics datasets available within the AAB.

## Supplementary information

**Additional file 1.** **Figure 1** First two principal components of the UK Biobank European subset (orange). Black dots denote the projection of the AAB ASD participants of European ancestry (n=698) onto the UK Biobank principal components. Blue dots denote the UK Biobank individuals selected as controls (n=5 per ASD participant). **Figure 2** Comparison of mean PGS +/- 95% confidence intervals for a) height and b) chronotype. Note that for chronotype, the input GWAS summary statistics were not fully independent due to overlap with UK Biobank participants. **Figure 3** Correlation between height PGS and measured height in a) adults and b) children. Correlation coefficient and p-values are provided in the bottom right corner of each plot. **Figure 4** Correlation between maternal and paternal ASD PGS. **Figure 5** Correlation between IQ PGS and measured IQ (WISC-IV in children; WASI matrix reasoning in adults) for a) all children, b) ASD only and stratified by IQ<70, c) SIB/UNR groups, d) parents. Correlation coefficient and p-values are provided in the bottom right corner of each plot. **Figure 6** Relationships between mean parent matrix reasoning IQ and child traits. a) Correlation between child’s age of ASD diagnosis and mean parent matrix reasoning IQ score (r=0.01, p=0.87). b) Density plot (n families = 249) for mean parent matrix reasoning IQ score, stratified by the presence of ID in their child. **Figure 7** Correlation between chronotype PGS and Children’s Sleep Habits Questionnaire (CSHQ) among a) all children, and b) the ASD group. Correlation coefficient and p-values are provided in the bottom right corner of each plot.

**Additional file 2.** **Table 1** Numbers of SNPs from each GWAS, and number remaining after QC (retaining SNPs in all of HapMap3 reference, UKB genotyping and AAB genotyping). **Table 2** SBayesR parRes output. **Table 3** Statistics for tests of comparison of PGS between groups (ASD, SIB, UNR, UKB). **Table 4** pTDT analysis results. **Table 5** Summary of statistics for PGS correlations. **Table 6** Variance explained by the PGS. **Table 7a** List of ASD/ID-related CNVs aggregated from ClinGen and DECIPHER. **b** List of ASD/ID/DD-related genes aggregated from Satterstrom et al. (2020) and DDD Study (2017) and overlapping with ENSEMBL hg19 gene set. Note that some genes are repeated as there are multiple transcripts that were considered for overlap. **Table 8a**: Identified CNVs in the AAB corresponding with CNVs reported by parents in the AAB medical history survey. Please note that this information is limited to that which was reported by parents, and is intended to be used to help assess replication in our pipeline. **b** Phenotypic correlates of called AAB CNVs overlapping ClinGen and DECIPHER CNVs. clingen_ddd.* provides start, end and widths of reference CNVs; critgene.* provides start and end of critical gene/s within reference CNVs; overlap_pcent refers to the % overlap of the called CNV, as a proportion of the ClinGen and/or DECIPHER-defined CNV. **c** Large CNVs (>1 Mb) identified within the AAB. **d** CNVs overlapping ASD/ID/DD-associated genes from Satterstrom et al. (2020) and DDD Study (2017). **Table 9 ** Overlap of Australian Autism Biobank (AAB) phenotypes with the Simons Simplex Collection (SSC) and Simons Foundation Powering Autism Research for Knowledge (SPARK).

## Data Availability

The datasets supporting the conclusions of this article are available by application to the Australian Autism Biobank within the Cooperative Research Centre for Living with Autism (Autism CRC): website at [15]. We also used data from the UK Biobank (website at [59]) within this study, under project number 12505. The individual-level data are available upon application to the UK Biobank.

## Abbreviations

AAB: Australian Autism Biobank
ADOS-2: Autism Diagnostic Observation Schedule-2
ADOS-G: Autism Diagnostic Observation Schedule-G
Autism CRC: Cooperative Research Centre for Living with Autism
ASD: Autism spectrum disorder. Predominantly used to denote the experimental group diagnosed with ASD, or queried for the condition
CSHQ: Children’s Sleep Habits Questionnaire
CNV: Copy-number variant
GWAS: Genome-wide association study
ID: Intellectual disability
IQ: Intelligence quotient
MSEL: Mullen Scales of Early Learning
PGS: Polygenic risk score
SIB: Sibling group
SNP: Single nucleotide polymorphism
SRS: Social Responsiveness Scale
SSP-2: Short Sensory Profile, version 2
UNR: Unrelated group without an ASD diagnosis
WASI: Wechsler Abbreviated Scale of Intelligence, 2nd edition
WISC-IV: Wechsler Intelligence Scale for Children, 4th edition
