# Supplementary material for: Analysis of common genetic variation and rare CNVs in the Australian Autism Biobank
Source: Mol Autism. 2021 Feb 10;12:12. doi: 10.1186/s13229-020-00407-5 (PMC7874616; doi:10.1186/s13229-020-00407-5)
Supplement: Supplementary file 1 — Additional file 1. Figure 1 First two principal components of the UK Biobank European subset (orange). Black dots denote the projection of the AAB ASD participants of European ancestry (n=698) onto the UK Biobank principal components. Blue dots denote the UK Biobank individuals selected as controls (n=5 per ASD participant). Figure 2 Comparison of mean PGS +/- 95% confidence intervals for a) height and b) chronotype. Note that for chronotype, the input GWAS summary statistics were not fully independent due to overlap with UK Biobank participants. Figure 3 Correlation between height PGS and measured height in a) adults and b) children. Correlation coefficient and p-values are provided in the bottom right corner of each plot. Figure 4 Correlation between maternal and paternal ASD PGS. Figure 5 Correlation between IQ PGS and measured IQ (WISC-IV in children; WASI matrix reasoning in adults) for a) all children, b) ASD only and stratified by IQ<70, c) SIB/UNR groups, d) parents. Correlation coefficient and p-values are provided in the bottom right corner of each plot. Figure 6 Relationships between mean parent matrix reasoning IQ and child traits. a) Correlation between child’s age of ASD diagnosis and mean parent matrix reasoning IQ score (r=0.01, p=0.87). b) Density plot (n families = 249) for mean parent matrix reasoning IQ score, stratified by the presence of ID in their child. Figure 7 Correlation between chronotype PGS and Children’s Sleep Habits Questionnaire (CSHQ) among a) all children, and b) the ASD group. Correlation coefficient and p-values are provided in the bottom right corner of each plot. [file 13229_2020_407_MOESM1_ESM.docx]

Supplementary Material

## UK Biobank controls


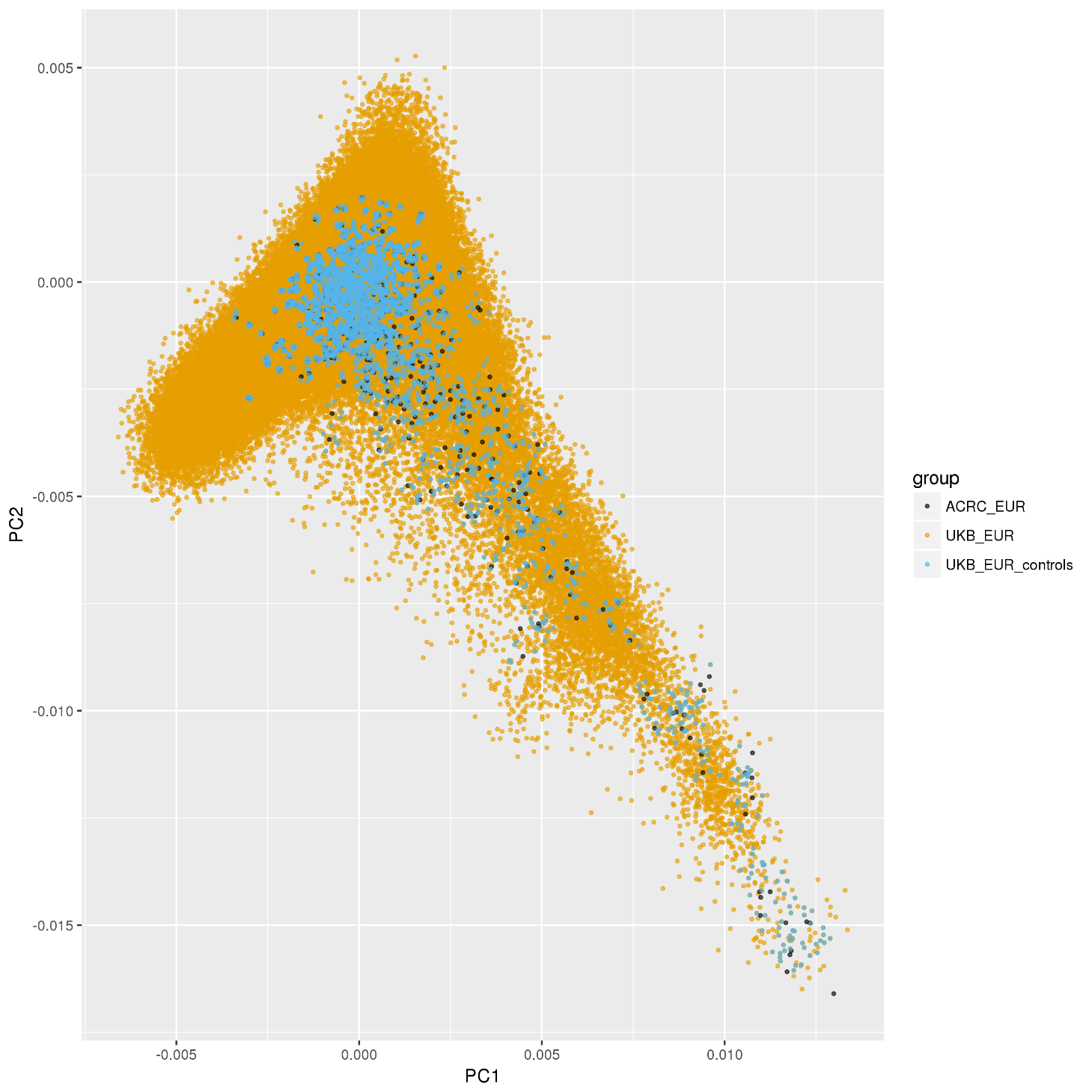


Supplementary Figure 1: First two principal components of the UK Biobank European subset (orange). Black dots denote the projection of the AAB ASD participants of European ancestry (n=698) onto the UK Biobank principal components. Blue dots denote the UK Biobank individuals selected as controls (n=5 per ASD participant).

## PGS group differences: height and chronotype


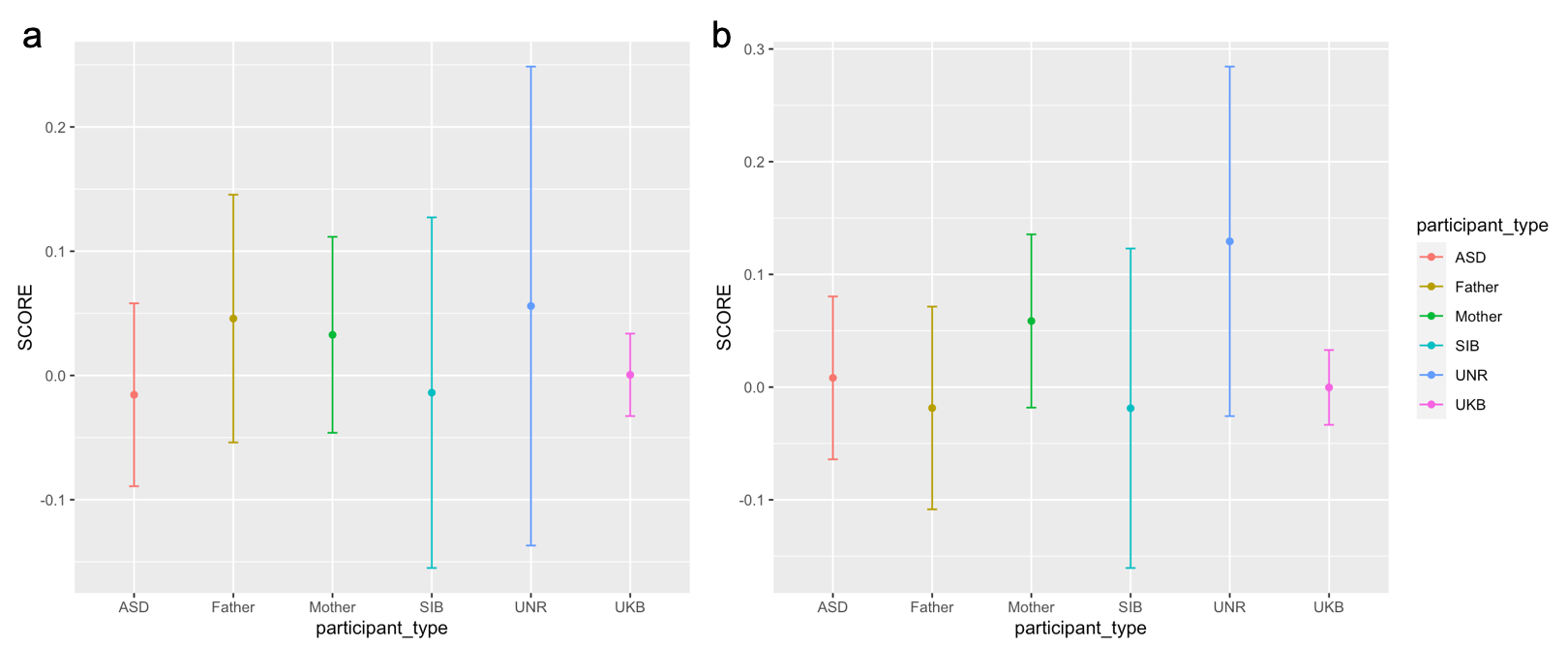


Supplementary Figure 2: Comparison of mean PGS +/- 95% confidence intervals for a) height and b) chronotype. Note that for chronotype, the input GWAS summary statistics were not fully independent due to overlap with UK Biobank participants.

## Height PGS: prediction in parents and children


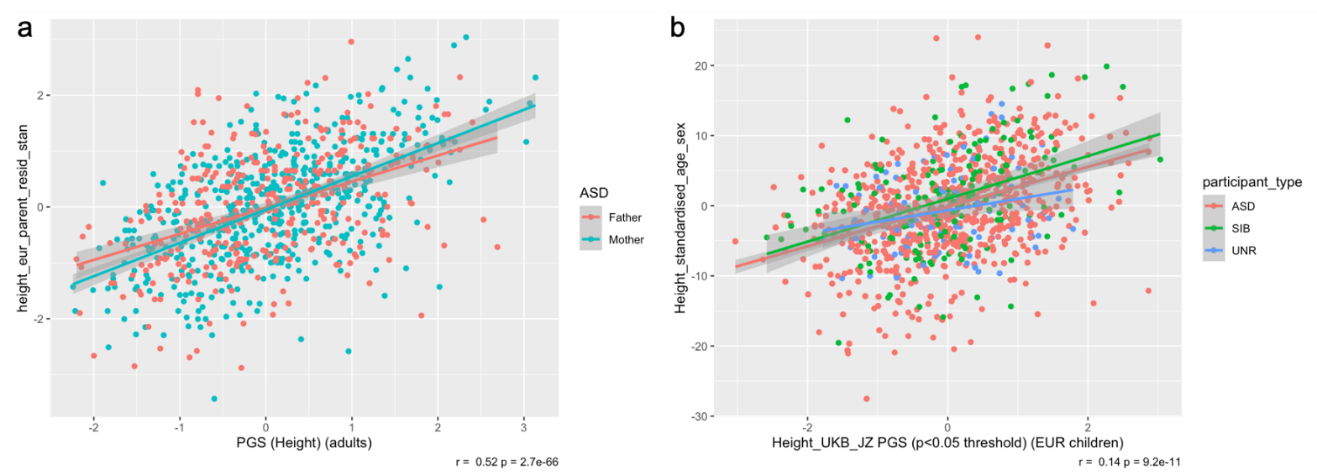


Supplementary Figure 3: Correlation between height PGS and measured height in a) adults and b) children. Correlation coefficient and p-values are provided in the bottom right corner of each plot.

## ASD PGS: correlation between parental PGS


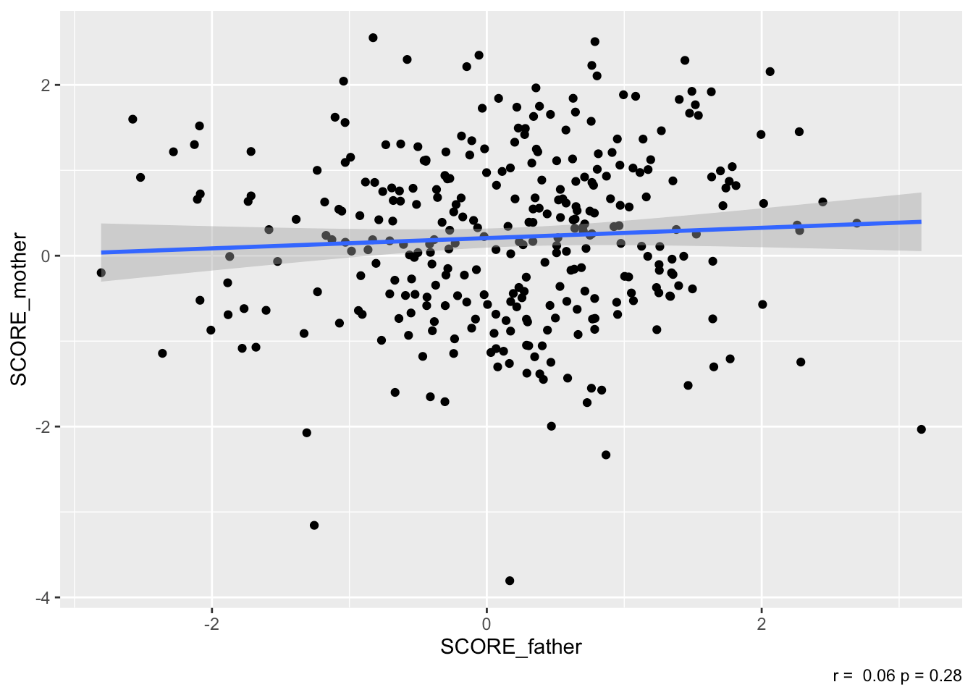


Supplementary Figure 4: Correlation between maternal and paternal ASD PGS

## IQ PGS: prediction in parents and children


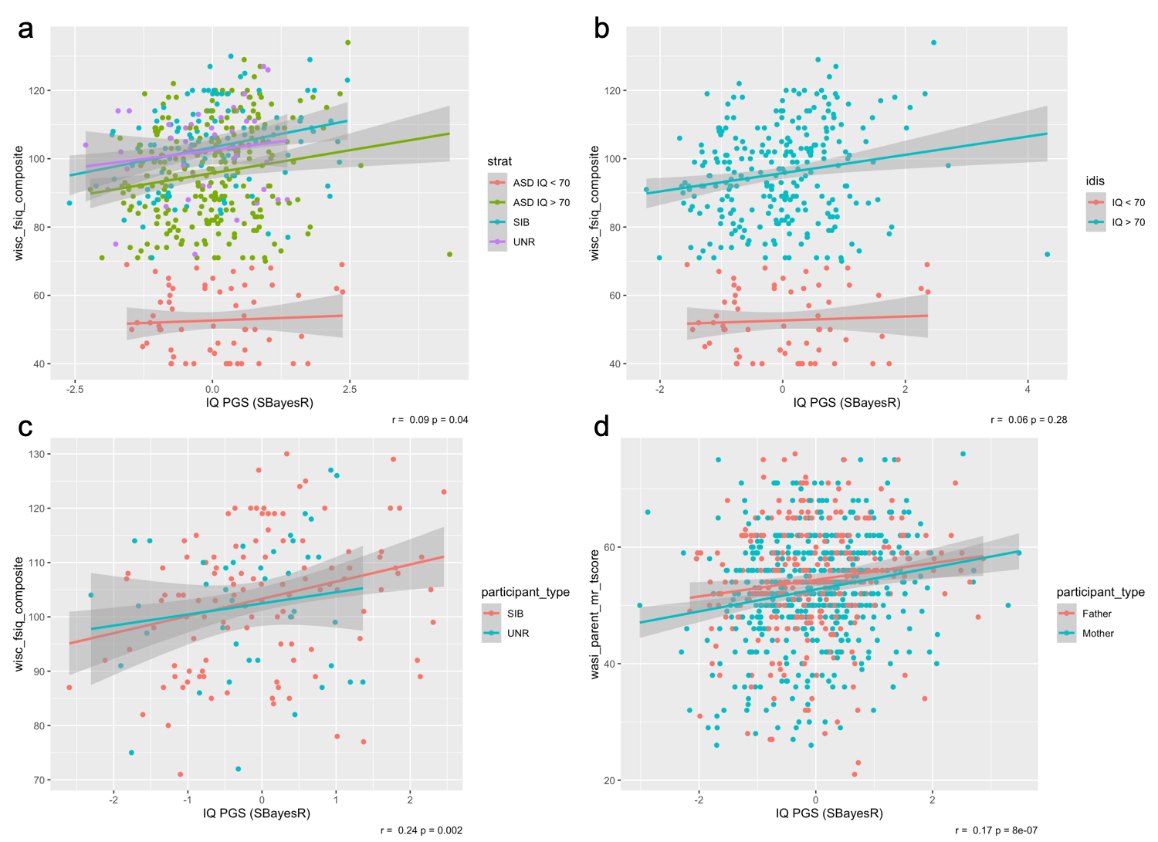


Supplementary Figure 5: Correlation between IQ PGS and measured IQ (WISC-IV in children; WASI matrix reasoning in adults) for a) all children, b) ASD only and stratified by IQ<70, c) SIB/UNR groups, d) parents. Correlation coefficient and p-values are provided in the bottom right corner of each plot.

## Parental matrix reasoning IQ and relationships with child’s traits


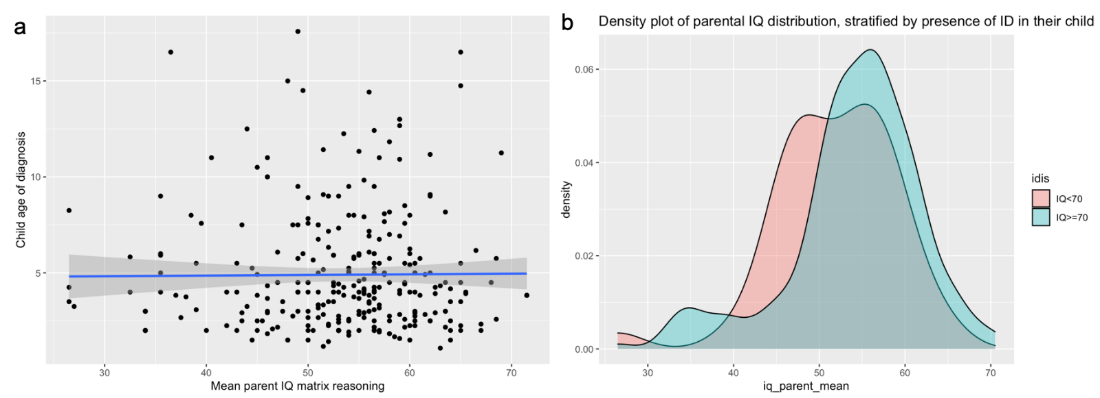


Supplementary Figure 6: Relationships between mean parent matrix reasoning IQ and child traits. a) Correlation between child’s age of ASD diagnosis and mean parent matrix reasoning IQ score (r=0.01, p=0.87). b) Density plot (n families = 249) for mean parent matrix reasoning IQ score, stratified by the presence of ID in their child.

## Chronotype PGS: correlation with Children’s Sleep Habits Questionnaire


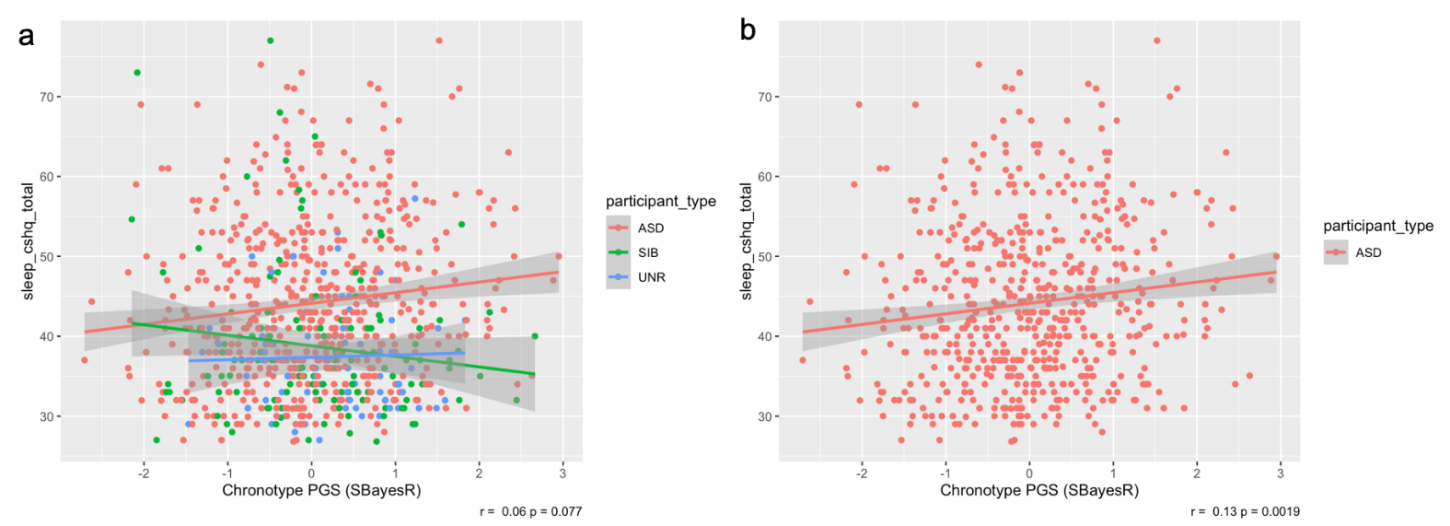


Supplementary Figure 7: Correlation between chronotype PGS and Children’s Sleep Habits Questionnaire (CSHQ) among a) all children, and b) the ASD group. Correlation coefficient and p-values are provided in the bottom right corner of each plot.
